# Supplementary material for: Implication of m6A mRNA Methylation in Susceptibility to Inflammatory Bowel Disease
Source: Epigenomes. 2020 Aug 3;4(3):16. doi: 10.3390/epigenomes4030016 (PMC8594712; doi:10.3390/epigenomes4030016)
Supplement: Supplementary file 1 [file epigenomes-04-00016-s001.pdf]

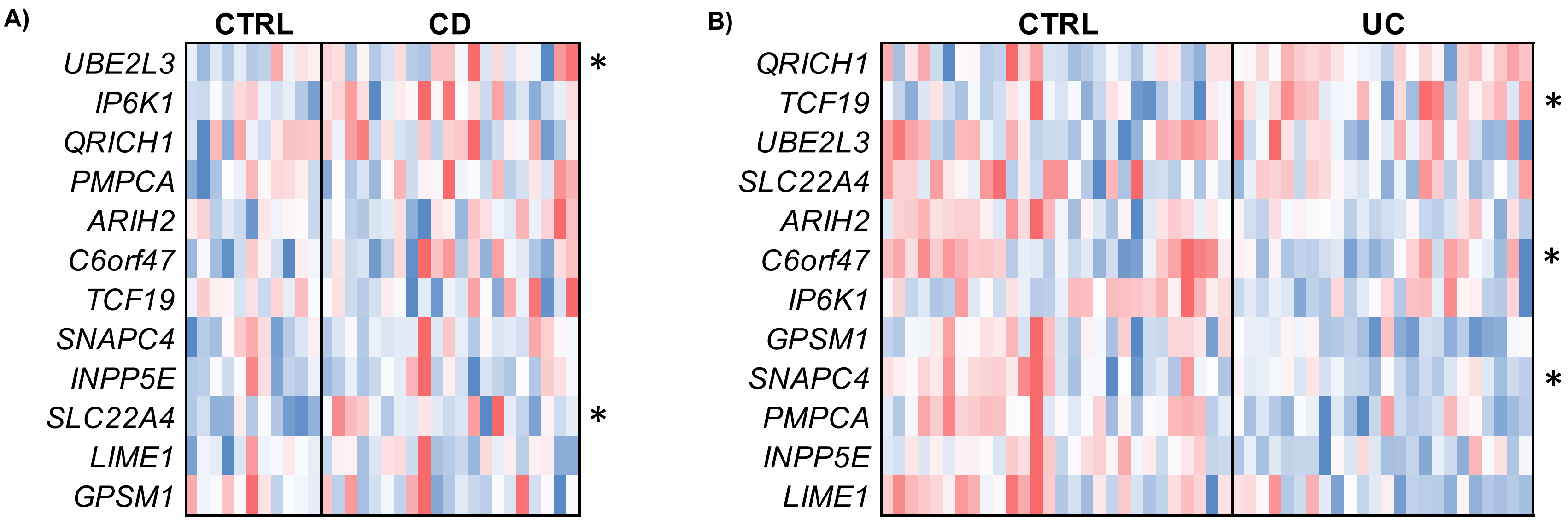

Supplementary Figure 1. Heatmaps of the differentially expressed genes harbouring a m6A-SNP A) CD and B) UC. Genes selected for further analyses are marked with an asterisk

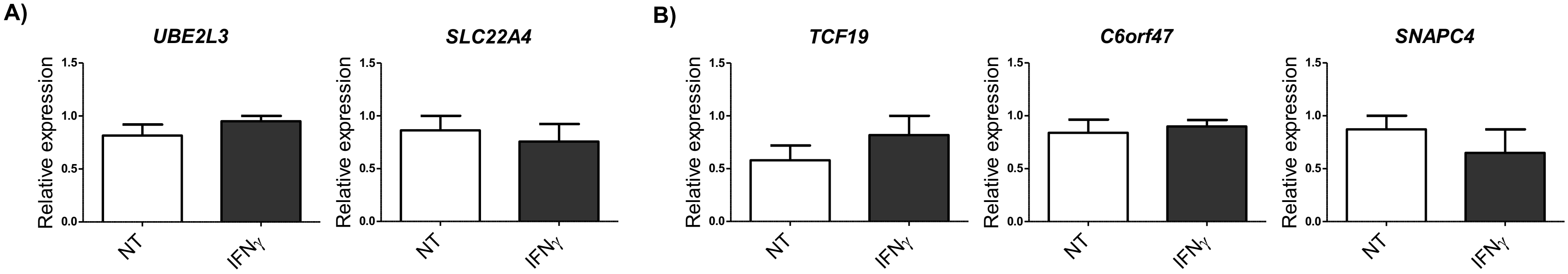

Supplementary Figure 2. Expression analyses of A) CD and B) UC candidate genes after 4h IFNγ treament. Data is represented as the mean and standard error of 4 independent experiments.

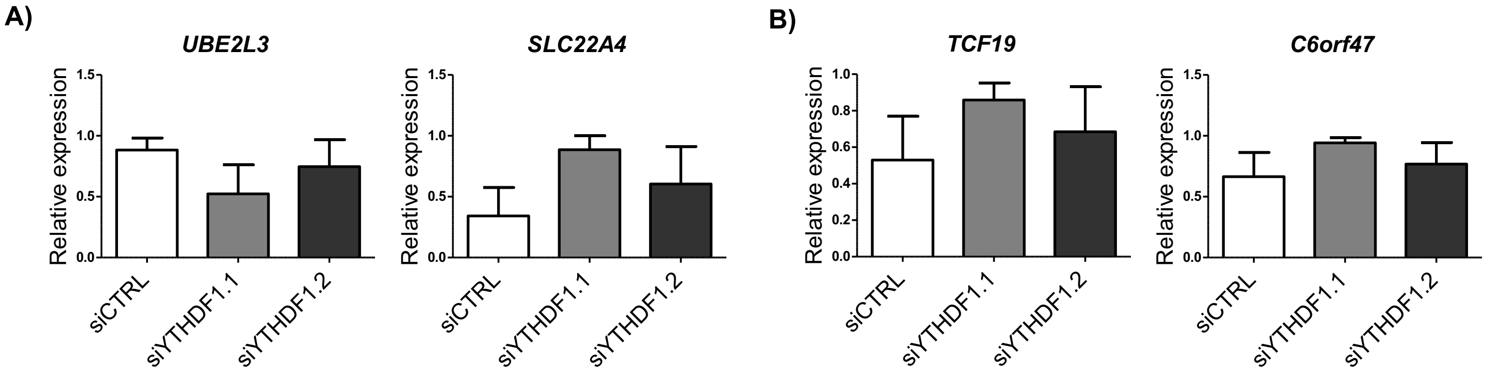

Supplementary Figure 3. Expression analyses of A) CD and B) UC candidate genes after YTHDF1 silencing. Values are represented as the mean and standard error of 3 independent experiments.

Supplementary table 1. Information about differentially expressed genes harbouring m6A-SNPs. Selected candidate genes for UC are highlited in blue and for CD in green.

	SNP associated			Differentially expressed in		Concordant in	
**Gene**	**IBD**	**CD**	**UC**	**CD**	**UC**	**CD**	**UC**
ARIH2	X				X		
C6orf47	X	X	X		X		YES
GPSM1	X				X		
INPP5E	X	X			X		
IP6K1	X	X			X		
LIME1	X	X			X		
PMPCA	X	X			X		
QRICH1	X				X		
SLC22A4	X	X		X		YES	
SNAPC4	X	X	X		X		YES
TCF19	X	X	X		X		YES
UBE2L3	X	X		X		YES	

Supplementary table 2. Specific primer pairs used for RT-QPCR expression analyses.

Gene	Forward primers	Reverse primers
METTL3	5'TCGAGAGCGAAATTTTTCAAC3'	5'GGAGATAGAGAGCCTTCTGAACC3'
METTL14	5'GAGTGTGTTTACGAAAATGGGGT3'	5'CCGTCTGTGCTACGCTTCA3'
WTAP	5'ACTGGCCTAAGACAGTCTGAAG3'	5'GTTGCTAGTCGCATTACAAGGA3'
YTHDF1	5'ACCTGTCCAGCTATTACCCG3'	5'TGGTGAGGTATGGAATCGGAG3'
UBE2L3	5'AGCTTGAAGAAATCCGCAAA3'	5'TGTGATCTTCGGTGGTTTGA3'
SLC22A4	5'GCCCAGGCGTTATATCATAGC3'	5'GCATGACCAGACCAATGGATAAG3'
TCF19	5' TCGGAGGAAATCTGTTCACC3'	5' GAGCGCTCACTGGGTACTTC3'
C6orf47	5'TGTGGGGCATCCCAAGACTAA3'	5'GCTCCCTAGCTGCTCAACTT3'
SNAPC4	5' GCAAGGAGTGGACAGAGGAG3'	5' CGTATTTGGCAACAGCTTGA3'
